# Association of Volume Status Assessed by Bioimpedance with BP in CKD

**DOI:** 10.34067/KID.0000000932

**Published:** 2025-08-14

**Authors:** Katherine Scovner Ravi, Enass Elsayed, Brendon L. Neuen, Glenn M. Chertow, Finnian R. Mc Causland

**Affiliations:** 1 Brigham and Women's Hospital, Boston, Massachusetts; 2 Harvard Medical School, Boston, Massachusetts; 3 The George Institute for Global Health, University of New South Wales, Sydney, Australia; 4 Departments of Medicine, Epidemiology and Population Health, and Health Policy, Stanford University School of Medicine, Stanford, California

**Keywords:** BP, CKD, hypertension

## Abstract

**Key Points:**

- Alhough hypervolemia contributes to hypertension, the association of BP with volume status measured by bioimpedance is unclear.
- Shorter and shortening vector length, proxies of hypervolemia, and volume expansion are associated with higher and increasing systolic BP.
- Bioimpedance-guided optimization of volume status may improve BP management among patients with CKD.

**Background:**

Hypertension is common among patients with CKD and is a risk factor for cardiovascular events and mortality. Although hypervolemia is a contributor to hypertension, the association of BP with biomarkers of volume among patients with CKD is unclear.

**Methods:**

Using data from 5384 patients in the Chronic Renal Insufficiency Cohort, we fit linear regression models to examine the association of vector length (bioimpedance proxy of volume) with systolic and diastolic BP. We used categorical analyses given evidence of nonlinear associations. We also assessed whether the change in vector length at 2 years from baseline was associated with changes in BP.

**Results:**

The mean age was 59±11 years; 44% were female; 43% were Black; mean systolic BP and eGFR were 129±21 mm Hg and 48±16 ml/min per 1.73 m^2^, respectively. The association of vector length with systolic BP was nonlinear; the lowest quartile of vector length (a proxy for hypervolemia) was associated with a 3.2 mm Hg (95% confidence interval, 1.1 to 5.3) higher systolic BP, compared with the third quartile. Compared with the third quartile over 2 years, the lowest quartile of change in vector length (a proxy for volume expansion) was associated with an increase in systolic BP (3.0 mm Hg; 95% confidence interval, 1.0 to 5.1). Diastolic BP was not associated with vector length.

**Conclusions:**

Shorter and shortening vector length were independently associated with higher and increasing systolic BP, respectively. Whether bioimpedance-guided optimization of volume status could improve BP management among patients with CKD requires further investigation.

## Visual Abstract

**Figure:**
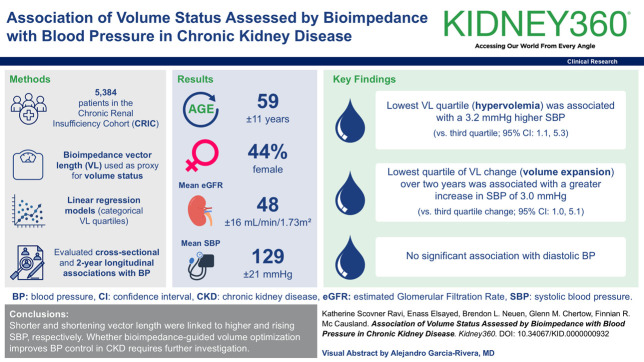

## Introduction

CKD affects 31 million Americans (approximately 1 in 7 adults)^1^ and is the ninth leading cause of death in the United States.^2^ Hypertension affects 60%–90% of patients with CKD, with a higher prevalence among those with more severe CKD.^3^ Furthermore, hypertension is an independent risk factor for cardiovascular events and all-cause mortality among patients with CKD.^4^

Many patients with advanced CKD develop hypervolemia due to a diminished capacity to excrete a daily salt load and subsequently develop hypertension.^5^ Acknowledging the limitations of physical examination in the assessment of volume status,^6^ several studies have assessed alternative methods of volume assessment in patients receiving hemodialysis.^7–11^ Bioelectrical impedance analysis (BIA) has received much attention due to its relatively low cost, portability, ease of use, and noninvasive nature.^12–14^ However, data evaluating BIA parameters and associations with BP in patients with nondialysis requiring CKD are limited.^14^

In this secondary analysis of the Chronic Renal Insufficiency Cohort (CRIC) study, we tested the hypotheses that (*1*) shorter vector length (a proxy for volume overload)^15,16^ would be associated with higher systolic and diastolic BP and (*2*) shortening of vector length over time (a proxy for volume expansion) would be associated with an increase in systolic and diastolic BP.

## Methods

### Study Design and Population

The CRIC is a prospective, multicenter study funded by the National Institute of Diabetes and Digestive and Kidney Diseases. The methods of cohort assembly and baseline clinical characteristics have previously been described.^17–19^ In brief, the study enrolled 3939 subjects from 2003 to 2008, an additional 1500 from 2013 to 2018, and then recruited an additional 125 Hispanic/Latino patients in a subsequent study phase.^20^ Patients were aged 21–74 years and had mild to moderate CKD (eGFR 20–70 ml/min per 1.73 m^2^). Approximately half had diabetes mellitus and half were female by design. Exclusion criteria included institutionalization, inability to provide consent, inability to participate in study procedures, New York Heart Association class 3 or 4 heart failure at baseline, cirrhosis, HIV and/or AIDS, pregnancy, history of dialysis, prior solid organ or bone marrow transplant, immunosuppressive therapy for primary kidney disease or vasculitis within the preceding 6 months, previous chemotherapy or alkylating agents for systemic cancer other than nonmelanoma skin cancer in the preceding 2 years, polycystic kidney disease, or participation in another research study. For this study, we restricted our analysis to the 5384 patients who had baseline BIA and BP data. Written informed consent was provided by all participants. The Institutional Review Boards at all participating centers approved and oversaw the CRIC study procedures.

### Exposure and Outcome Variables

The primary exposure of interest was vector length (Z), measured by single-frequency BIA, indexed to height (H) in meters. Z/H was calculated from the raw measurements of resistance (R) and reactance (Xc), where R represents the opposition to the flow of an alternating current through ionic solutions and Xc is the capacitance produced by interfaces across tissues (*e.g*., cell membranes), according to the following formula: $|Z/H|=\sqrt{\left[{\left(R/H\right)}^{2}+{\left(X_{c}/H\right)}^{2}\right]}$.^21,22^ A shorter vector length reflects a higher degree of soft tissue volume. We also examined the change in vector length (baseline to year 2) as a secondary exposure of interest. BIA measurements were performed using a single-frequency Quantum II bioelectrical impedance analyzer (RLJ Systems) at baseline and at subsequent annual visits.

Vector length was preferentially chosen as the exposure of interest as it is based on the measured impedance parameters of resistance and reactance and therefore independent of model assumptions related to body weight, such as percent fluid overload.^23^ In this respect, vector length reflects overall tissue hydration and cellular mass, rather than a specific body “compartment.” Reporting of vector length is also consistent with prior publications from CRIC,^24,25^ as well as *post hoc* analyses of the Frequent Hemodialysis Network trials,^26^ allowing comparisons to be made more easily. For ease of interpretability, previously reported reference values are approximately 412±39 Ω/m for non-Hispanic White patients, 331±42 Ω/m for non-Hispanic Black patients, and 345±42 Ω/m for Mexican-American patients, using data from National Health and Nutrition Examination Survey.^23^ The mean vector length from a prior cohort of approximately 3000 US patients receiving maintenance hemodialysis was 300±70 Ω/m.^15^

The primary outcome of interest was systolic BP (mm Hg). We also assessed diastolic BP as a secondary outcome. As per the CRIC study protocol, BP measurements were obtained after participants sat quietly at rest for 5 minutes. An aneroid sphygmomanometer was used, and one of four cuff sizes (pediatric, regular, large, or thigh) was selected based on the participant's arm circumference. Participants were asked to abstain from coffee, tea, alcohol, cigarettes, or vigorous exercise for 30 minutes before their physical examination. Researchers completed training sessions on BP measurement methods and were certified to examine BP based on their performance on a written exam.^27^

### Statistical Analyses

We examined continuous variables graphically and recorded data as mean (±SD) for normally distributed variables or median (with 25th to 75th percentile range) for non-normally distributed variables. We examined categorical variables by frequency distribution and recorded data as proportions. We compared characteristics across quartiles of the vector length using tests for trend based on linear regression, chi-squared trend test, and the Cuzick nonparametric trend test, as appropriate for the data distribution.

We used restricted cubic spline to assess for nonlinear associations of vector length with systolic and diastolic BP in the fully adjusted models. As nonlinearity was evident, we assessed the association of categories of vector length with systolic and diastolic BP using unadjusted and adjusted linear regression. Model 1 adjusted for age, sex, designated race, and body mass index (BMI; weight in kg/height in m^2^). Model 2 additionally adjusted for history of heart failure, diabetes, coronary artery disease, peripheral vascular disease, and stroke as well as for hematocrit, serum phosphate, and albumin concentrations, smoking status, and eGFR based on the CKD Epidemiology Collaboration (CKD-EPI) equation.^28^ Model 3 additionally adjusted for the number of BP medications prescribed (*i.e*., angiotensin-converting enzyme inhibitors, angiotensin receptor blockers, *α*-blockers, *α*-2 adrenergic agonists, *β* adrenergic antagonists, calcium channel blockers, potassium-sparing diuretics, loop diuretics, thiazide and thiazide-like agents, and direct vasodilators) prescribed, log-transformed urine protein per 24 hours, and urine sodium excretion. As a sensitivity analysis, we also ran a version of the fully adjusted model excluding eGFR and 24-hour urine sodium to assess the potential for overadjustment. We tested for effect modification according to baseline age, sex, designated race, eGFR, and log-transformed urine protein excretion per 24 hours through the inclusion of cross-product terms in the fully adjusted model. We also assessed the association of the change in vector length from baseline to 2 years with the change in systolic and diastolic BP. These models were additionally adjusted for the baseline vector length (Z/H) and baseline BP.

We considered two-tailed *P* values <0.05 as statistically significant. We conducted analyses using Stata MP (version 18.0, Stata Corp., College Station, TX).

## Results

### Baseline Characteristics

Baseline characteristics of included and excluded patients are provided in Supplemental Table 1. In brief, those excluded tended to be older, less likely to be White, have greater frequency of comorbid diseases, higher baseline eGFR, diastolic BP, number of antihypertensive medications, and have shorter vector length.

**Figure 1:**
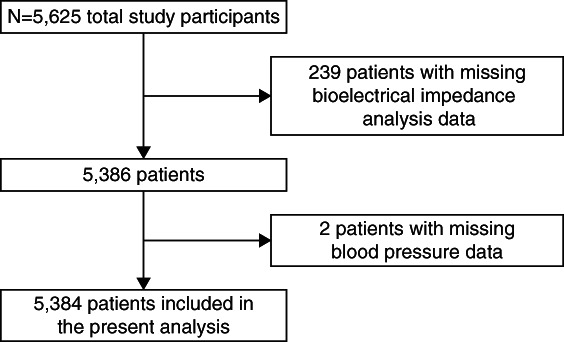
**Consort diagram.** Flow diagram showing selection of study participants from the CRIC cohort. Of 5625 total participants, 5384 (96%) had both bioimpedance vector length and BP measurements available at baseline and were included in the analysis. CRIC, Chronic Renal Insufficiency Cohort.

Vector length and BP data were available at baseline for 5384 (96%) patients in the CRIC study (Figure 1). The mean age was 59±11 years, 44% were female, and 43% were non-Hispanic Black, 41% were non-Hispanic White, and 16% were other. Patients in lower quartiles of vector length were younger; were more likely to be male; had higher BMI; were more likely to have a history of heart failure, diabetes, ischemic heart disease, and peripheral vascular disease; had higher hematocrit and lower serum phosphate and albumin concentrations; were less likely to be current smokers; had lower eGFR; were prescribed more categories of BP medications; and had higher urine protein and higher urine sodium excretion (Table 1).

**Table 1:** Baseline characteristics according to category of vector length

Characteristic		Quartile 1 *n*=1346	Quartile 2 *n*=1346	Quartile 3 *n*=1346	Quartile 4 *n*=1346	*P* Value
Vector length, Ω/m		203±26	252±11	295±14	371±41	<0.001
Age, yr		58±10	60±11	60±11	60±11	=0.002
Female, *n*(%)		188 (14.0%)	328 (24.4%)	659 (49.0%)	1191 (88.5%)	<0.001
**Race, *n*(%)**						=0.50
Non-Hispanic Black		619 (46.0%)	577 (42.9%)	564 (41.9%)	561 (41.7%)	
Non-Hispanic White		521 (38.7%)	563 (41.8%)	549 (40.8%)	565 (42.0%)	
Other		206 (15.3%)	206 (15.3%)	233 (17.3%)	220 (16.3%)	
BMI, kg/m^2^		37±8	33±7	31±7	28±6	<0.001
History of heart failure, *n*(%)		159 (11.8%)	118 (8.8%)	106 (7.9%)	81 (6.0%)	<0.001
History of diabetes, *n*(%)		908 (67.5%)	752 (55.9%)	650 (48.3%)	443 (32.9%)	<0.001
History of ischemic heart disease, *n*(%)		348 (25.9%)	290 (21.5%)	273 (20.3%)	197 (14.6%)	<0.001
History of peripheral vascular disease, *n*(%)		125 (9.3%)	88 (6.5%)	73 (5.4%)	55 (4.1%)	<0.001
History of stroke, *n*(%)		134 (10.0%)	147 (10.9%)	130 (9.7%)	127 (9.4%)	=0.44
Hematocrit, %		37.8±5.3	38.6±5.4	38.1±5.0	37.2±4.4	<0.001
Phosphate, mg/dl		3.7±0.7	3.7±0.7	3.7±0.6	3.8±0.6	=0.003
Albumin, g/dl		3.8±0.5	4.0±0.4	4.0±0.4	4.0±0.4	<0.001
Current smoker, *n*(%)		131 (9.7%)	176 (13.1%)	176 (13.1%)	187 (13.9%)	=0.002
eGFR (CKD-EPI), ml/min per 1.73 m^2^		47±15	49±16	49±15	49±16	=0.03
Urine protein excretion, g/24 h	0.5 [0.1–2.1]	0.2 [0.1–0.9]	0.2 [0.1–0.7]	0.1 [0.1–0.4]		<0.001
Urine sodium excretion, mmol/24 h	87±31.92	87±33	80±34	73±36		<0.001
No. of antihypertensive medications, *n*(%)	3.2±1.6	2.7±1.6	2.5±1.5	2.2±1.5		<0.001
Systolic BP, mm Hg	133±21	129±21	127±21	125±22		<0.001
Diastolic BP, mm Hg	73±13	72±13	71±12	70±12		<0.001

Results are presented as mean±SD, or median [25th–75th percentiles] for continuous variables.

We compared characteristics across quartiles of the vector length using tests for trend based on linear regression, Chi-squared trend test, and the Cuzick nonparametric trend test, as appropriate for the data distribution. BMI, body mass index; CKD-EPI, CKD Epidemiology Collaboration.

### Association of Vector Length with Systolic BP

The mean baseline vector length was 280±67 Ω/m. The mean baseline systolic BP was 129±21 mm Hg. Spline analysis demonstrated a nonlinear association of vector length with systolic BP (*P* < 0.001; Supplemental Figure 1). The lowest quartile of vector length was associated with 3.2 mm Hg (95% confidence interval [CI], 1.1 to 5.3) higher systolic BP, relative to the third quartile in the fully adjusted model (Table 2). The sensitivity model excluding eGFR and 24-hour urine sodium yielded similar but slightly stronger associations (Supplemental Table 2).

**Table 2:** Association of vector length with systolic BP

Model	Difference in Systolic BP in mm Hg (95% CI)			
	Quartile 1	Quartile 2	Quartile 3	Quartile 4
Unadjusted	5.3 (3.7 to 6.9)	1.5 (−0.1 to 3.1)	Ref	−2.0 (−3.6 to −0.4)
Model 1	8.8 (7.0 to 10.6)	3.5 (1.9 to 5.1)	Ref	−4.4 (−6.2 to −2.7)
Model 2	6.3 (4.1 to 8.4)	2.5 (0.5 to 4.4)	Ref	−1.9 (−4.0 to 0.2)
Model 3	3.2 (1.1 to 5.3)	1.1 (−0.8 to 2.9)	Ref	−0.9 (−2.9 to 1.1)

Model 1 adjusted for age, sex, race, and body mass index. Model 2 additionally adjusted for history of heart failure, diabetes, coronary artery disease, peripheral vascular disease, and stroke; hematocrit, serum phosphate, and serum albumin levels; smoking status; and eGFR based on the CKD Epidemiology Collaboration equation. Model 3 additionally adjusted for the number of BP medication categories prescribed, log transformed urine protein per 24 hours, and 24-hour urine sodium excretion. BMI, body mass index; CI, confidence interval.

The association of vector length with systolic BP differed according to age and eGFR (*P*-interaction < 0.001 for both) in the fully adjusted model. Specifically, the association of the lowest quartile (relative to the third quartile) of vector length with systolic BP appeared to be more potent for those aged >61 than for those ≤61 years (3.7 [95% CI, 0.5 to 7.0] and 2.4 [95% CI, −0.3 to 5.2] mm Hg, respectively). Regarding eGFR, the association of the lowest quartile (versus third quartile) of vector length with systolic BP appeared to be more potent among those with eGFR >48.2 than for those with eGFR ≤48.2 ml/min per 1.73 m^2^ (4.9 [95% CI, 1.6 to 8.2] and 2.4 [95% CI, −0.3 to 5.1] mm Hg, respectively). There was no suggestion of differential associations according to sex, race, or log-transformed 24-hour urine protein (*P*-interaction 0.18, 0.13, and 0.92, respectively).

### Association of Change in Vector Length with Change in Systolic BP

Data on change in vector length and change in BP from baseline to 2 years were available for *n*=3005 participants. Each decrease in vector length of 50 Ω/m was associated with an increase in systolic BP of 2.9 mm Hg (95% CI, 1.9 to 3.8) in the fully adjusted model. The lowest quartile of vector length change was associated with 3.0 mm Hg (95% CI, 1.0 to 5.1) greater increase in systolic BP, relative to the third quartile in the fully adjusted model (Table 3).

**Table 3:** Association of difference in vector length over 2 years with difference in systolic BP

Model	Difference in Systolic Blood Pressure (95% CI) in mm Hg Per 50 Ω/m decrease vector length	Quartile 1	Quartile 2	Quartile 3	Quartile 4
Unadjusted	2.3 (1.4 to 3.1)	4.0 (2.0 to 6.1)	−0.0 (−2.0 to 2.0)	Ref	−1.4 (−3.4 to 0.6)
Model 1	3.1 (2.2 to 4.0)	3.9 (1.9 to 5.9)	0.2 (−1.7 to 2.2)	Ref	−2.6 (−4.6 to −0.6)
Model 2	3.1 (2.2 to 4.0)	3.6 (1.6 to 5.6)	0.6 (−1.4 to 2.6)	Ref	−3.1 (−5.1 to −1.1)
Model 3	2.9 (1.9 to 3.8)	3.0 (1.0 to 5.1)	0.1 (−1.9 to 2.1)	Ref	−3.4 (−5.5 to −1.4)

All models adjusted for baseline vector length and systolic BP. Model 1 adjusted for age, sex, race, and body mass index. Model 2 additionally adjusted for history of heart failure, diabetes, coronary artery disease, peripheral vascular disease, and stroke; hematocrit, serum phosphate, and serum albumin levels; smoking status; and eGFR based on the CKD Epidemiology Collaboration equation. Model 3 additionally adjusted for the number of BP medication categories prescribed, log transformed urine protein per 24 hours, and 24-hour urine sodium excretion. BMI, body mass index; CI, confidence interval.

### Association of Vector Length with Diastolic BP

The mean diastolic BP was 71±13 mm Hg. Spline analysis demonstrated a nonlinear association of vector length with diastolic BP (*P* < 0.01; Supplemental Figure 2). In fully adjusted categorical analyses, the lowest quartile of vector length was associated with 0.9 mm Hg (95% CI, −0.4 to 2.1) higher diastolic BP, compared with the third quartile (Supplemental Table 3).

### Association of Change in Vector Length with Change in Diastolic BP

Each decrease in vector length of 50 Ω/m was associated with a mean increase in diastolic BP of 0.3 mm Hg (95% CI, −0.2 to 0.8) in the fully adjusted model. The lowest quartile of vector length change was associated with 0.3 mm Hg (95% CI, −0.8 to 1.5) greater increase in diastolic BP, relative to the third quartile in the fully adjusted model (Supplemental Table 4).

## Discussion

In this *post hoc* analysis of data from the CRIC study, we observed an inverse association between vector length and systolic BP. In other words, shorter vector length (a proxy for volume overload) was associated with higher systolic BP. We also observed that a shortening of vector length (a proxy for volume expansion) from baseline to year 2 was associated with an increase in systolic BP, while a lengthening of vector length was associated with a decrease in systolic BP. Neither vector length at baseline nor change in vector length was associated with diastolic BP or change in diastolic BP, respectively.

Volume overload is common in advanced CKD and is believed to be partly due to reduced filtration of sodium, as well as an inappropriate suppression of tubular sodium reabsorption, predisposing to volume expansion and higher BP.^29,30^ Our observed association of shorter vector length with higher systolic BP is consistent with this hypothesis, as shorter vector length is believed to be reflective of extracellular volume overload.^31^ The absence of a similar association with diastolic BP aligns with our understanding that systolic BP is more responsive to changes in stroke volume and cardiac output, which are in turn responsive to blood volume.^32^ Conversely, diastolic BP is believed to be more influenced by peripheral vascular resistance^32,33^ because of arteriolar tone or vascular compliance.^34,35^ In this respect, endothelial dysfunction with reduced endothelium-dependent vasodilation, as well as medial calcification and increased arterial stiffness,^36–38^ may contribute.^34,35^ Other possible contributions may relate to inflammation,^39^ reflecting the complex interplay among volume status, vascular resistance, and BP in patients with CKD. Although likely oversimplified, this complexity has been described by some in relation to two axes—volume and BP.^40,41^ The correlation between hypervolemia and hypertension and their opposites is straight-forward to understand, but acknowledging that hypertension+hypovolemia (*e.g*., vasoconstriction) and hyotension+hypervolemia (*e.g*., heart failure) can coexist is also important. This further highlights the need to better understand the potential differential responsiveness of systolic and diastolic BP to volume status and to the pharmacologic interventions commonly used to treat hypertension.

Objective measures for assessing volume status may be more reliable than traditional clinical assessment.^42^ However, there are limited published data on the utility of objective measures of tissue hydration among patients with nondialysis requiring CKD. Of the studies that exist in this population, one review identified 11 cohorts and found an association of indices of volume overload with mortality, cardiovascular outcomes, and CKD progression.^43^ Among patients enrolled in the Study of Heart and Kidney Protection With Empagliflozin^44^ trial in whom BIA data were obtained (*n*=660), empagliflozin reduced both BIA-derived proxies of volume overload and systolic BP.^14,45^ In another study performed in patients with mild-to-moderate CKD (mean eGFR 60±21 ml/min per 1.73 m^2^), as little as 1L excess extracellular water measured by BIA was associated with the development of left ventricular hypertrophy and diastolic dysfunction.^46^ Such small amounts of excess volume may be difficult to identify on physical examination, supporting the potential for BIA to identify patients with CKD at increased risk for heart failure, in addition to guiding the use of dietary modification, diuretics, and other medications.^14^

Our study has several strengths. The CRIC study was relatively large in size, and participants were diverse by age, sex, designated race and ethnicity, as well as biopsy-proven or presumed primary kidney disease. We adjusted for a wide range of clinical characteristics that might have confounded the associations between vector length and BP. Leveraging repeated bioimpedance and BP measurements in over 5000 patients with CKD allowed us to conduct both cross-sectional and longitudinal analyses. The latter are of particular note, as we observed that changes in vector length were associated with changes in BP, supporting the hypothesis that volume status should be considered a modifiable risk factor among patients with established CKD. However, there were several notable limitations. First, CRIC was not designed to test the association of BIA with BP, and despite the use of adjusted models, the possibility of residual confounding remains. Second, vector length by BIA may be confounded by changes in body composition unrelated to volume expansion, particularly in longitudinal analyses. We did not adjust for intercurrent events (*e.g*., hospitalized heart failure, stroke, peripheral arterial events, including amputation) or inflammatory conditions which could lead to muscle wasting and sharply reduce tissue reactance.^35^ Third, patients with CKD and New York Heart Association class 3 and 4 heart failure were excluded from CRIC; these patients may have the most to gain from more nuanced assessment of volume status. Furthermore, it is likely that patients with CKD willing to participate in a multiyear long cohort study differ considerably in ways that were not measured (including factors related to social vulnerability). As there are no widely accepted thresholds of vector length for defining volume overload, our estimates are based on the CRIC distribution, which limits generalizability of the findings to the broader CKD population.

In conclusion, we observed an association between vector length and systolic BP among patients with CKD, confirming the links between volume overload and volume expansion, systolic BP, and severity of hypertension. Several novel antihypertensive agents are currently under study in patients with CKD, including mineralocorticoid receptor antagonists,^47^ aldosterone synthase inhibitors,^48^ and endothelin receptor antagonists,^49^ and two renal denervation devices^50,51^ were recently approved for use in patients whose hypertension remains uncontrolled despite lifestyle interventions and medical therapy. As BIA is increasingly accessible, portable, and noninvasive,^35^ future studies of established and/or novel therapies in patients with CKD might incorporate BIA to identify relatively subtle differences in volume status, ultimately aiming to optimize management of hypertension in this high-risk population.

## Supplementary Material

## Data Availability

Original data generated for the study are or will be made available in a public access repository upon publication. Published Material. CRIC. https://journals.lww.com/jasn/fulltext/2003/07002/the_chronic_renal_insufficiency_cohort__cric_.18.aspx. doi: 10.1097/01.ASN.0000070149.78399.CE.

## Declarative Statements

This study includes clinical experimentation and received Institutional Review Board or Ethics Committee approval. All patients provided written informed consent.

## Supplemental Material

Supplemental Table 1. Baseline characteristics comparing included and excluded patients.

Supplemental Table 2. Association of vector length with systolic BP in fully adjusted model excluding eGFR and 24-hour urine sodium.

Supplemental Table 3. Association of vector length with diastolic BP.

Supplemental Table 4. Association of difference in vector length over 2 years with difference in diastolic BP.

Supplemental Figure 1. Association of vector length with diastolic BP.
