# Supplementary material for: Association of Volume Status Assessed by Bioimpedance with BP in CKD
Source: Kidney360. 2025 Aug 14;7(1):157–63. doi: 10.34067/KID.0000000932 (PMC12889916; doi:10.34067/KID.0000000932)
Supplement: Supplementary file 1 [file kidney360-7-157-s001.pdf]

ASN American Society of Nephrology
JASN Journal of the American Society of Nephrology
KIDNEY360 Accessing Our World From Every Angle
CJASN Clinical Journal of the American Society of Nephrology

ASN Journal Disclosure Form

As per ASN journal policy, I have disclosed any financial relationships or commitments I have held in the past 36 months as included below. I have listed my Current Employer below to indicate there is a relationship requiring disclosure. If no relationship exists, my Current Employer is not listed.

G. Chertow reports the following:
Employer: Stanford University School of Medicine; Consultancy: Akebia, Alebund, Ardelyx, AstraZeneca, CalciMedica, Miromatrix, Panoramic, Sanifit, Toku, Unicycive, Vertex; Ownership Interest: Ardelyx, CloudCath, Durect, Eliaz Therapeutics, Outset, Renibus, Unicycive; Research Funding: NIDDK, NIAID, CSL Behring; Advisory or Leadership Role: Board of Directors, Satellite Healthcare, Co-Editor, Brenner & Rector's The Kidney (Elsevier); and Other Interests or Relationships: DSMB service: NIDDK, George Institute, Aethlon, Bayer, Mineralys, ReCor.

I understand that the information above will be published within the journal article, if accepted, and that failure to comply and/or to accurately and completely report the potential financial conflicts of interest could lead to the following: 1) Prior to publication, article rejection, or 2) Post-publication, sanctions ranging from, but not limited to, issuing a correction, reporting the inaccurate information to the authors' institution, banning authors from submitting work to ASN journals for varying lengths of time, and/or retraction of the published work.

Name: Glenn M. Chertow
Manuscript ID: K360-2025-000217R1
Manuscript Title: Association of Volume Status Assessed by Bioimpedance with Blood Pressure in Chronic Kidney Disease
Date of Completion: June 2, 2025
Disclosure Updated Date: January 22, 2025

ASN American Society of Nephrology
JASN Journal of the American Society of Nephrology
KIDNEY360 Accessing Our World From Every Angle
CJASN Clinical Journal of the American Society of Nephrology

ASN Journal Disclosure Form

As per ASN journal policy, I have disclosed any financial relationships or commitments I have held in the past 36 months as included below. I have listed my Current Employer below to indicate there is a relationship requiring disclosure. If no relationship exists, my Current Employer is not listed.

E. Elsayed has nothing to disclose.

I understand that the information above will be published within the journal article, if accepted, and that failure to comply and/or to accurately and completely report the potential financial conflicts of interest could lead to the following: 1) Prior to publication, article rejection, or 2) Post-publication, sanctions ranging from, but not limited to, issuing a correction, reporting the inaccurate information to the authors' institution, banning authors from submitting work to ASN journals for varying lengths of time, and/or retraction of the published work.

Name: Enass Sayed Elsayed
Manuscript ID: (K360-2025-000217R2)
Manuscript Title: ("Association of Volume Status Assessed by Bioimpedance with Blood Pressure in Chronic Kidney Disease")
Date of Completion: August 8, 2025
Disclosure Updated Date: May 27, 2025

ASN
American Society of Nephrology
JASN
Journal of the American Society of Nephrology
KIDNEY360
Accessing Our World From Every Angle
CJASN
Clinical Journal of the American Society of Nephrology

ASN Journal Disclosure Form

As per ASN journal policy, I have disclosed any financial relationships or commitments I have held in the past 36 months as included below. I have listed my Current Employer below to indicate there is a relationship requiring disclosure. If no relationship exists, my Current Employer is not listed.

F. Mc Causland reports the following:
Employer: Brigham and Women's Hospital; Consultancy: Aquapass; GlaxoSmithKline; Zydus Therapeutics Inc.; Research Funding: Research Funding from Novartis, Lexicon, AstraZeneca, NIH, paid directly to his institution; Honoraria: Travel Support from Bayer; Speakers Bureau: Bayer and Global Learning Collaborative; and Other Interests or Relationships: Expert witness fees from Rubin-Anders scientific; his spouse reports consulting fees from Vera Therapeutics and Alexion; serving on a DSMB for AstraZeneca.

I understand that the information above will be published within the journal article, if accepted, and that failure to comply and/or to accurately and completely report the potential financial conflicts of interest could lead to the following: 1) Prior to publication, article rejection, or 2) Post-publication, sanctions ranging from, but not limited to, issuing a correction, reporting the inaccurate information to the authors' institution, banning authors from submitting work to ASN journals for varying lengths of time, and/or retraction of the published work.

Name: Finnian R. Mc Causland
Manuscript ID: K360-2025-000217R1
Manuscript Title: "Association of Volume Status Assessed by Bioimpedance with Blood Pressure in Chronic Kidney Disease,
Date of Completion: June 3, 2025
Disclosure Updated Date: April 21, 2025

ASN American Society of Nephrology
JASN Journal of the American Society of Nephrology
KIDNEY360 Accessing Our World From Every Angle
CJASN Clinical Journal of the American Society of Nephrology

ASN Journal Disclosure Form

As per ASN journal policy, I have disclosed any financial relationships or commitments I have held in the past 36 months as included below. I have listed my Current Employer below to indicate there is a relationship requiring disclosure. If no relationship exists, my Current Employer is not listed.

B. Neuen reports the following:
Employer: The George Institute for Global Health, UNSW, Sydney, Australia; Royal North Shore Hospital, Sydney, Australia.; Consultancy: AstraZeneca, Alexion, Bayer, Boehringer and Ingelheim, Travere, Otsuka, Vera Therapeutics.; Research Funding: Astrazeneca, Bayer, Menarini; Honoraria: AstraZeneca, Bayer, Boehringer and Ingelheim, CSL-Behring, CSL-Seqirus, Novo Nordisk.; and Speakers Bureau: AstraZeneca, Boehringer and Ingelheim, Bayer, Novo Nordisk.

I understand that the information above will be published within the journal article, if accepted, and that failure to comply and/or to accurately and completely report the potential financial conflicts of interest could lead to the following: 1) Prior to publication, article rejection, or 2) Post-publication, sanctions ranging from, but not limited to, issuing a correction, reporting the inaccurate information to the authors' institution, banning authors from submitting work to ASN journals for varying lengths of time, and/or retraction of the published work.

Name: Brendon Lange Neuen
Manuscript ID: K360-2025-000217R2
Manuscript Title: Association of Volume Status Assessed by Bioimpedance with Blood Pressure in Chronic Kidney Disease
Date of Completion: July 1, 2025
Disclosure Updated Date: June 11, 2025

ASN American Society of Nephrology
JASN Journal of the American Society of Nephrology
KIDNEY360 Accessing Our World From Every Angle
CJASN Clinical Journal of the American Society of Nephrology

ASN Journal Disclosure Form

As per ASN journal policy, I have disclosed any financial relationships or commitments I have held in the past 36 months as included below. I have listed my Current Employer below to indicate there is a relationship requiring disclosure. If no relationship exists, my Current Employer is not listed.

K. Ravi reports the following:
Employer: Brigham and Women's Hospital; and Ownership Interest: Halo LLC (spouse).

I understand that the information above will be published within the journal article, if accepted, and that failure to comply and/or to accurately and completely report the potential financial conflicts of interest could lead to the following: 1) Prior to publication, article rejection, or 2) Post-publication, sanctions ranging from, but not limited to, issuing a correction, reporting the inaccurate information to the authors' institution, banning authors from submitting work to ASN journals for varying lengths of time, and/or retraction of the published work.

Name: Katherine Scovner Ravi
Manuscript ID: K360-2025-000217R1
Manuscript Title: Association of Volume Status Assessed by Bioimpedance with Blood Pressure in Chronic Kidney Disease
Date of Completion: June 5, 2025
Disclosure Updated Date: June 5, 2025
