# Supplementary material for: Association of Volume Status Assessed by Bioimpedance with BP in CKD
Source: Kidney360. 2025 Aug 14;7(1):157–63. doi: 10.34067/KID.0000000932 (PMC12889916; doi:10.34067/KID.0000000932)
Supplement: Supplementary file 2 [file kidney360-7-157-s002.pdf]

SUPPLEMENTAL MATERIAL TABLE OF CONTENTS:

Supplemental Table 1. Baseline characteristics comparing included and excluded patients

Supplemental Table 2. Association of vector length with systolic blood pressure in fully adjusted model excluding eGFR and 24-hour urine sodium

Supplemental Table 3. Association of vector length with diastolic blood pressure

Supplemental Table 4. Association of difference in vector length over 2 years with difference in diastolic blood pressure

Supplemental Figure 1. Association of vector length with systolic blood pressure

Supplemental Figure 2. Association of vector length with diastolic blood pressure

Supplemental Table 1. Baseline characteristics comparing included and excluded patients

	exclude= 0<br>n=5384	exclude= 1<br>n=241	
Vector length, Ω/m	280 ± 67	179 ± 163	p=0.03
Age, years	59 ± 11	62 ± 9	p<0.001
Female	2366 (43.9%)	91 (37.8%)	p=0.06
Race, n(%)			P<0.001
Non-Hispanic White	2198 (40.8%)	78 (32.4%)	
Non-Hispanic Black	2321 (43.1%)	101 (41.9%)	
Other	865 (16.1%)	62 (25.7%)	
Body Mass Index, kg/m^2	32.3 ± 7.6	32.8 ± 8.3	p=0.30
History of heart failure, n(%)	464 (8.6 %)	78 (32.4%)	p<0.001
History of diabetes, n(%)	2753 (51.1%)	141 (58.5%)	p=0.03
History of ischemic heart disease, n(%)	1108 (20.6%)	98 (40.7%)	p<0.001
History of peripheral vascular disease, n(%)	341 (6.3 %)	34 (14.1%)	p<0.001
History of stroke, n(%)	538 (10.0%)	37 (15.4%)	P<0.01
Hematocrit, %	37.9 ± 5.0	38.0 ± 4.6	p=0.95
Phosphate, mg/dL	3.7 ± 0.7	3.8 ± 0.7	p=0.14
Albumin, g/dL	3.9 ± 0.5	3.9 ± 0.4	p=0.38
Current smoker, n(%)	670 (12.4%)	37 (15.4%)	p=0.18
eGFR (CKD-EPI), ml/min/1.73 m^2	48 ± 16	51 ± 16	p=0.03
Urine protein excretion, g/24 hours	0.2 [0.1, 0.9]	0.2 [0.1, 1.0]	p=0.21
Urine sodium excretion, mmol/24 hours	81.5 ± 34.1	74.5 ± 26.7	p=0.05
No. of anti-hypertensive medications, n(%)	2.6 ± 1.6	3.0 ± 1.7	p=0.002
Systolic BP, mmHg	129 ± 21	127 ± 21	p=0.37
Diastolic BP, mmHg	71 ± 13	69 ± 12	p=0.01

Results are presented as mean ± standard deviation, or median [25th-75th percentiles] for continuous variables.

Abbreviations: BMI, body mass index; eGFR, estimated glomerular filtration rate; CKD-EPI, Chronic Kidney Disease Epidemiology Collaboration.

Supplemental Table 2. Association of vector length with systolic blood pressure in fully adjusted model excluding eGFR and 24-hour urine sodium

	Difference in systolic BP in mmHg (95%CI)			
Model	Quartile 1	Quartile 2	Quartile 3	Quartile 4
Sensitivity Model	3.5 (1.4, 5.6)	1.3 (-0.6, 3.2)	Ref	-0.9 (-2.9, 1.1)

Model adjusted for age, gender, race, body mass index (BMI), history of heart failure, diabetes, coronary artery disease, peripheral vascular disease, and stroke, hematocrit, serum phosphate, and serum albumin levels, smoking status, the number of blood pressure medication categories prescribed, and log transformed urine protein per 24 hours.

Supplemental Table 3. Association of vector length with diastolic blood pressure

Model	Difference in diastolic blood pressure (95%CI) in mmHg<br>Per 50 Ω/m lower vector length	Quartile 1	Quartile 2	Quartile 3	Quartile 4
Unadjusted	0.8 (0.5, 1.0)	1.9 (0.9, 2.8)	1.2 (0.2, 2.1)	Ref	-1.0 (-1.9, -0.0)
Model 1	0.0 (-0.3, 0.4)	0.4 (-0.7, 1.4)	0.3 (-0.6, 1.2)	Ref	0.0 (-1.0, 1.0)
Model 2	0.5 (0.1, 1.0)	2.2 (0.9, 3.5)	1.4 (0.3, 2.5)	Ref	-0.2 (-1.4, 1.0)
Model 3	-0.0 (-0.5, 0.4)	0.9 (-0.4, 2.1)	0.6 (-0.5, 1.7)	Ref	0.2 (-1.0, 1.4)

Model 1 adjusted for age, gender, race, and body mass index (BMI). Model 2 additionally adjusted for history of heart failure, diabetes, coronary artery disease, peripheral vascular disease, and stroke, hematocrit, serum phosphate, and serum albumin levels, smoking status, and estimated glomerular filtration rate based on the CKD Epidemiology Collaboration (EPI) equation. Model 3 additionally adjusted for the number of blood pressure medication categories prescribed, log transformed urine protein per 24 hours, and 24 hour urine sodium excretion.

Supplemental Table 4. Association of difference in vector length over 2 years with difference in diastolic blood pressure

	Difference in diastolic blood pressure (95%CI) in mmHg				
Model	Per 50 Ω/m decrease vector length	Quartile 1	Quartile 2	Quartile 3	Quartile 4
Unadjusted	0.5 (0.0, 1.0)	0.7 (-0.4, 1.8)	0.5 (-0.6, 1.5)	Ref	-0.2 (-1.2, 0.9)
Model 1	0.2 (-0.3, 0.7)	0.3 (-0.8, 1.4)	0.4 (-0.6, 1.5)	Ref	0.1 (-1.0, 1.1)
Model 2	0.3 (-0.2, 0.8)	0.5 (-0.6, 1.6)	0.3 (-0.8, 1.3)	Ref	0.1 (-1.0, 1.2)
Model 3	0.3 (-0.2, 0.8)	0.3 (-0.8, 1.5)	0.1 (-1.0, 1.2)	Ref	0.0 (-1.1, 1.1)

All models adjusted for baseline vector length and diastolic blood pressure. Model 1 adjusted for age, gender, race, and body mass index (BMI). Model 2 additionally adjusted for history of heart failure, diabetes, coronary artery disease, peripheral vascular disease, and stroke, hematocrit, serum phosphate, and serum albumin levels, smoking status, and estimated glomerular filtration rate based on the CKD Epidemiology Collaboration (EPI) equation. Model 3 additionally adjusted for the number of blood pressure medication categories prescribed, log transformed urine protein per 24 hours, and 24 hour urine sodium excretion.

SUPPLEMENTAL FIGURE LEGEND

Supplemental Figure 1. Association of vector length with systolic blood pressure
The solid black line represents the association of vector length with sysstolic blood pressure, adjusted for age, gender, race, body mass index (BMI), history of heart failure, diabetes, coronary artery disease, peripheral vascular disease, and stroke as well as for hematocrit, serum phosphate, and serum albumin levels, smoking at time of assessment, estimated glomerular function based on the CKD Epidemiology Collaboration (EPI) equation, the number of blood pressure medication categories prescribed, log transformed urine protein per 24 hours, and urine sodium levels; the dashed lines are the 95% confidence intervals; the histogram in the background represents the frequency of patients with various vector lengths.

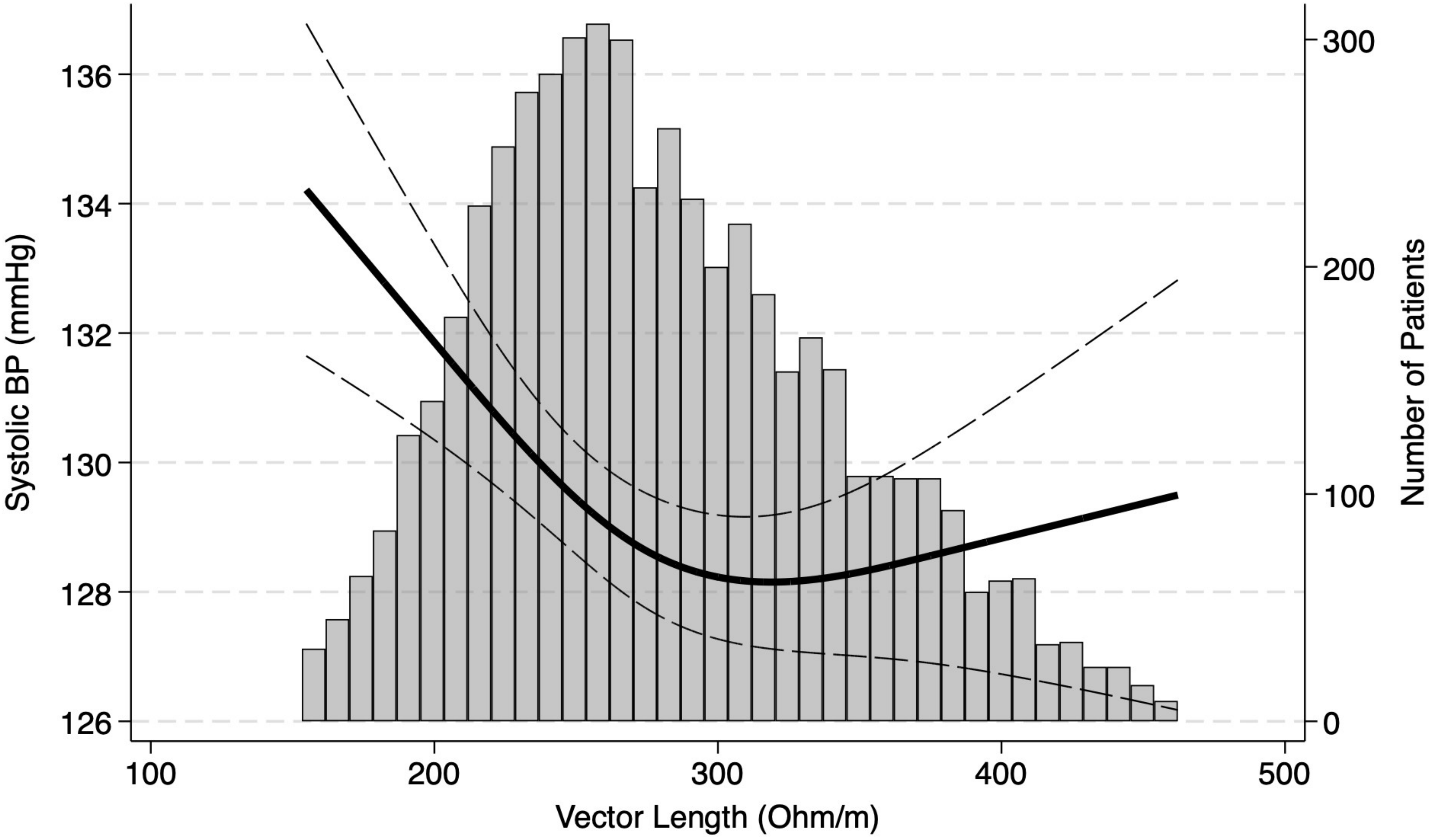

Systolic BP (mmHg)
136
134
132
130
128
126
Number of Patients
300
200
100
0
100
200
300
400
500
Vector Length (Ohm/m)

Supplemental Figure 2. Association of vector length with diastolic blood pressure
The solid black line represents the association of vector length with diastolic blood pressure, adjusted for age, gender, race, body mass index (BMI), history of heart failure, diabetes, coronary artery disease, peripheral vascular disease, and stroke as well as for hematocrit, serum phosphate, and serum albumin levels, smoking at time of assessment, estimated glomerular function based on the CKD Epidemiology Collaboration (EPI) equation, the number of blood pressure medication categories prescribed, log transformed urine protein per 24 hours, and urine sodium levels; the dashed lines are the 95% confidence intervals; the histogram in the background represents the frequency of patients with various vector lengths.

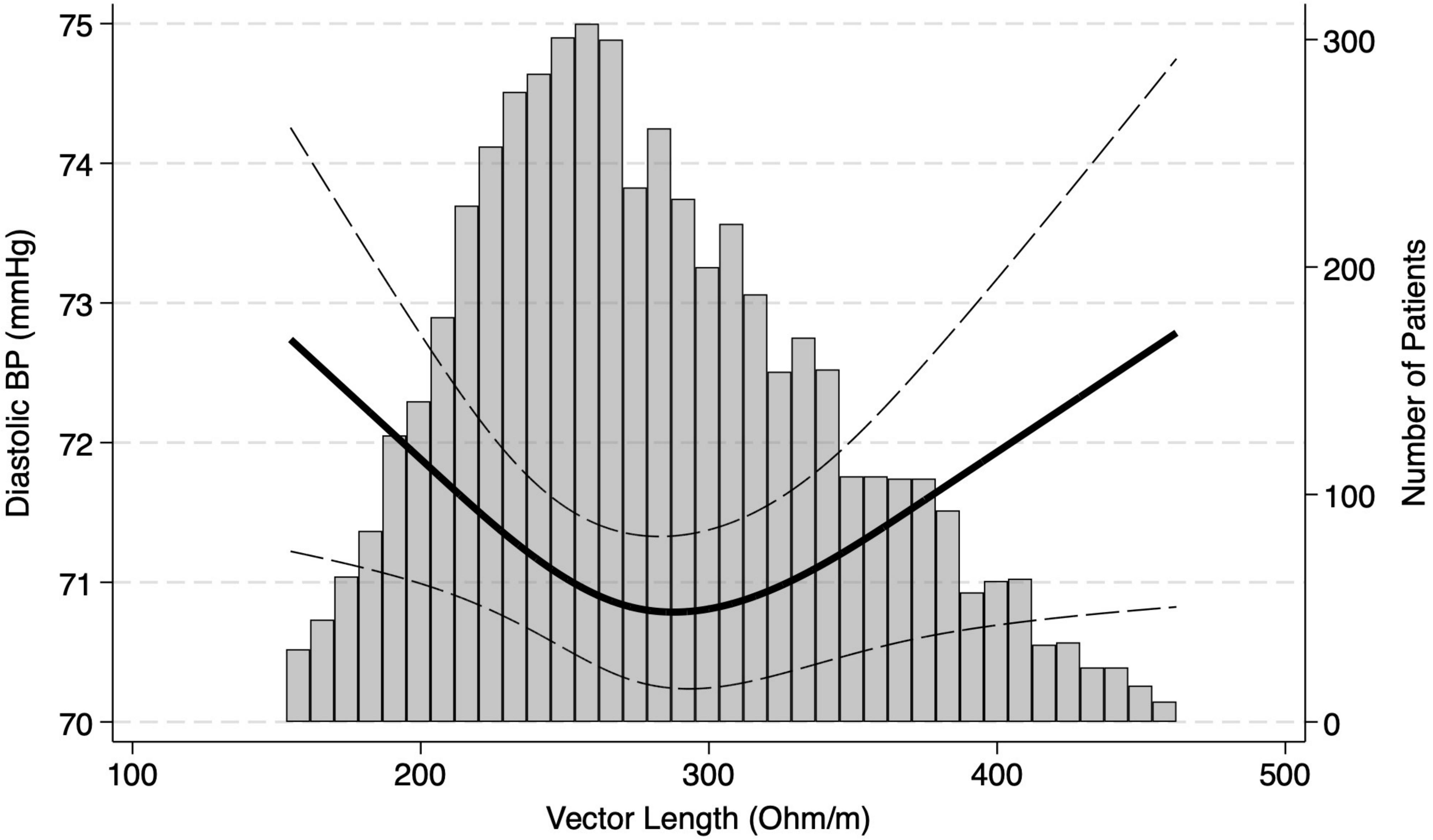

Diastolic BP (mmHg)
75
74
73
72
71
70
Number of Patients
300
200
100
0
100
200
300
400
500
Vector Length (Ohm/m)
